# Managing Granulomatous–Lymphocytic Interstitial Lung Disease in Common Variable Immunodeficiency Disorders: e-GLILDnet International Clinicians Survey

**DOI:** 10.3389/fimmu.2020.606333

**Published:** 2020-11-26

**Authors:** Annick A. J. M. van de Ven, Tiago M. Alfaro, Alexandra Robinson, Ulrich Baumann, Anne Bergeron, Siobhan O. Burns, Alison M. Condliffe, Børre Fevang, Andrew R. Gennery, Filomeen Haerynck, Joseph Jacob, Stephen Jolles, Marion Malphettes, Véronique Meignin, Tomas Milota, Joris van Montfrans, Antje Prasse, Isabella Quinti, Elisabetta Renzoni, Daiana Stolz, Klaus Warnatz, John R. Hurst

**Affiliations:** ^1^ Departments of Internal Medicine and Allergology, Rheumatology and Clinical Immunology, University Medical Center Groningen, Netherlands; ^2^ Pneumology Unit, Centro Hospital e Universitário de Coimbra, Coimbra, Portugal and Centre of Pneumology, Faculty of Medicine, University of Coimbra, Coimbra, Portugal; ^3^ UCL Respiratory, University College London, London, United Kingdom; ^4^ Department of Paediatric Pulmonology, Allergy and Neonatology, Hannover Medical School, Hannover, Germany; ^5^ Université de Paris, Assistance Publique Hôpitaux de Paris (APHP), Hôpital Saint Louis, Paris, France; ^6^ Institute of Immunity and Transplantation, University College London, Dept of Immunology, Royal Free London NHS Foundation Trust, London, United Kingdom; ^7^ Department of Infection, Immunity and Cardiovascular Diseases, University of Sheffield Medical School, Sheffield, United Kingdom; ^8^ Centre for Rare Disorders and Section of Clinical Immunology and Infectious Diseases, Oslo University Hospital, Oslo, Norway; ^9^ Translational and Clinical Research Institute, Newcastle University and Great North Children’s Hospital, Newcastle upon Tyne, United Kingdom; ^10^ Department of Pediatric Pulmonology and Immunology, Centre for Primary Immune deficiency Ghent, PID research lab, Ghent University Hospital, Belgium; ^11^ Centre for Medical Image Computing, University College London, London, United Kingdom; ^12^ Immunodeficiency Centre for Wales, University Hospital of Wales, Cardiff, United Kingdom; ^13^ Department of Clinical Immunology, Hôpital Saint-Louis, Assistance Publique Hôpitaux de Paris (APHP), Université Paris Diderot, Paris, France; ^14^ Department of Pathology, Hôpital Saint-Louis, Assistance Publique Hôpitaux de Paris (APHP), Paris, France; ^15^ Department of Immunology, Second Faculty of Medicine Charles University and Motol University Hospital, Prague, Czech Republic; ^16^ Department of Pediatric Immunology and Infectious Diseases, University Medical Center Utrecht, Utrecht, Netherlands; ^17^ Department of Pulmonology, Hannover Medical School and DZL BREATH, and Fraunhofer Institute for Toxicology and Experimental Medicine, Hannover, Germany; ^18^ Department of Molecular Medicine, Sapienza University of Rome, Rome, Italy; ^19^ Interstitial Lung Disease Unit, Royal Brompton Hospital, London, United Kingdom; ^20^ Clinic for Respiratory Medicine and Pulmonary Cell Research, University Hospital Basel, Basel, Switzerland; ^21^ Department of Rheumatology and Clinical Immunology, Medical Center - University of Freiburg, Faculty of Medicine, University of Freiburg, Freiburg, Germany; ^22^ Center for Chronic Immunodeficiency, Medical Center - University of Freiburg, Faculty of Medicine, University of Freiburg, Freiburg, Germany

**Keywords:** CVID, GLILD, interstitial lung disease, e-GLILDnet, diagnosis, follow-up, treatment

## Abstract

**Background:**

Granulomatous–lymphocytic interstitial lung disease (GLILD) is a rare, potentially severe pulmonary complication of common variable immunodeficiency disorders (CVID). Informative clinical trials and consensus on management are lacking.

**Aims:**

The European GLILD network (e-GLILDnet) aims to describe how GLILD is currently managed in clinical practice and to determine the main uncertainties and unmet needs regarding diagnosis, treatment and follow-up.

**Methods:**

The e-GLILDnet collaborators developed and conducted an online survey facilitated by the European Society for Immunodeficiencies (ESID) and the European Respiratory Society (ERS) between February–April 2020. Results were analyzed using SPSS.

**Results:**

One hundred and sixty-one responses from adult and pediatric pulmonologists and immunologists from 47 countries were analyzed. Respondents treated a median of 27 (interquartile range, IQR 82–maximum 500) CVID patients, of which a median of 5 (IQR 8–max 200) had GLILD. Most respondents experienced difficulties in establishing the diagnosis of GLILD and only 31 (19%) had access to a standardized protocol. There was little uniformity in diagnostic or therapeutic interventions. Fewer than 40% of respondents saw a definite need for biopsy in all cases or performed bronchoalveolar lavage for diagnostics. Sixty-six percent used glucocorticosteroids for remission-induction and 47% for maintenance therapy; azathioprine, rituximab and mycophenolate mofetil were the most frequently prescribed steroid-sparing agents. Pulmonary function tests were the preferred modality for monitoring patients during follow-up.

**Conclusions:**

These data demonstrate an urgent need for clinical studies to provide more evidence for an international consensus regarding management of GLILD. These studies will need to address optimal procedures for definite diagnosis and a better understanding of the pathogenesis of GLILD in order to provide individualized treatment options. Non-availability of well-established standardized protocols risks endangering patients.

## Introduction

Common variable immunodeficiency (CVID) disorders are the most prevalent symptomatic primary immunodeficiency (PID) conditions, characterized by hypogammaglobulinemia together with an increased susceptibility to infections and/or, in a minority of patients, clinically significant immune dysregulation (1). Immune dysregulation includes autoimmune and autoinflammatory conditions, lymphoproliferative disease and can result in both solid organ and hematologic malignancies. With generally efficacious administration of immunoglobulin substitution and antimicrobial agents, immune dysregulation now imposes the heaviest burden on morbidity and mortality of CVID patients. The term “CVID” was in 2009 redefined by the International Union of Immunological Societies Expert Primary Immunodeficiency Committee into “CVID disorders”, emphasizing the heterogeneity of this collection of inborn errors of immunity (2). The number of potential distinct entities within this group remains unknown and although novel monogenic forms are still being identified, the majority of cases is assumed to be of complex and polygenic inheritance (3, 4).

Lung involvement is very common in CVID disorders and typically has two not mutually exclusive entities: structural abnormalities such as bronchial wall thickening, air trapping and bronchiectasis that can arise as complications of recurrent bronchopulmonary infections; and interstitial lung disease (ILD) including parenchymal and interstitial abnormalities (ground glass opacities, nodules and consolidation) that are considered to be driven by intrinsic CVID-related immune dysregulation. This ILD in CVID disorders is commonly referred to as granulomatous-lymphocytic interstitial lung disease or GLILD. The estimated prevalence of GLILD in CVID disorders is around 15% and may already be present in childhood CVID disorders (5–7).

GLILD was defined by a UK Consortium as “a distinct clinico-radio-pathological ILD occurring in patients with CVID disorders, associated with a lymphocytic infiltrate and/or granuloma in the lung, and in whom other conditions have been considered and where possible excluded”, recognizing that this GLILD is “usually seen in the context of multisystem granulomatous/inflammatory involvement” (8). This definition of GLILD was unanimously supported by all participants. Agreement scores on other aspects of GLILD diagnosis were lower: for instance, 47% agreed that GLILD patients need to be symptomatic. The report went on to describe that diagnostic evaluation should include spirometry (96% consensus), lung volumes (91%), gas transfer (100%), flexible bronchoscopy to exclude infection (83%), surgical lung biopsy (83%) and computed tomography (CT, all respondents). Consensus was defined that lung biopsy specimens should be stained for CD3, CD4, CD8, CD20, for the presence of bacteria including *Mycobacteria* and for fungi, and for clonality to exclude lymphoma (8).

The pathogenesis of GLILD remains unclear and is considered to be heterogeneous. Histologic studies reveal infiltration of both T- and B-lymphocytes, partly leading to the formation of tertiary lymphoid structures. Increased concentrations of local and serum B-cell activating factor (BAFF) possibly drive B-lymphocyte hyperplasia (9).

GLILD is a rare condition and therefore there is a lack of robust scientific evidence, especially about therapeutics. There are currently no published randomized controlled trials or prospective cohort studies investigating the effects of immunomodulatory treatments as many difficulties arise in recruiting an adequate number of participants. A systematic review is included in this collection (Lamers et al., this issue). Current investigations are exclusively observational studies; this is problematic as they are unable, by design, to include randomization and concealment of allocation (10).

The first step in GLILD treatment consists of optimization of CVID disorders management, including Ig replacement therapy (IgRT). Antimicrobial prophylaxis may be used in a proportion of patients, with initiation of immunosuppressive therapy given that IgRT alone is not generally effective to treat GLILD (11–15). As with many inflammatory conditions, corticosteroids are often the first choice for remission induction in GLILD. Corticosteroids often result in an improvement in GLILD, however following prednisolone therapy of 1–4 months a widely heterogeneous response was observed, as many patients do not exhibit any improvements in PFTs or had disease flares upon tapering of corticosteroid medication (16). Collectively, these findings define the need for re-evaluation of corticosteroid monotherapy as first-line treatment.

Regarding second-line immunosuppressive therapy, various drugs have been employed. Small case series (17) and single case reports (18–21) show a potential effect of rituximab as monotherapy. Rituximab is also documented to be used in combination with azathioprine (21–28), 6-MP (22, 29) or mycophenolate mofetil (22, 28, 30), supporting a role for B-lymphocytes in the pathogenesis of GLILD. Other therapies include conventional disease modifying anti-rheumatic drugs (cDMARD), such as cyclophosphamide and methotrexate, however, current evidence is limited and lacks scientific support through a lack of controlled clinical trials (31). We are not aware of any reports using novel anti-fibrotics used in fibrotic ILD such as nintedanib and pirfenidone.

The scarcity and low level of quality of scientific literature on GLILD highlights knowledge gaps in essential aspects of GLILD, including pathogenesis, diagnostic evaluation and therapy. Since GLILD is a rare disease, these data can only be obtained by means of constructive, multicenter and multidisciplinary collection and collaboration.

With this aim, the e-GLILDnet was established in 2019 as a Clinical Research Collaboration of the European Respiratory Society (https://www.ersnet.org/research/e-glildnet—a-european-granulomatous-lymphocytic-interstitial-lung-disease-network; twitter: @glildnet) (32). A first workstream of this group was to conduct an online questionnaire among treating physicians of which the results are described here.

## Methods

An online questionnaire was distributed to members of the European Respiratory Society (ERS) and European Society for Immunodeficiencies (ESID) between February 19 and April 30, 2020, and promoted on Social Media.

The questionnaire was developed by the e-GLILDnet collaborators and pretested. Questions were designed by authors with experience in immunology (AV, KW) and pulmonology (TA, JH) and previous experience in designing online questionnaires (KW) (33) Multiple rounds of revision within this author group and subsequently the entire e-GLILDnet team followed. Final adjustments were made after testing an online pilot version. The questionnaire was distributed (in English), and comprised 35 combined open/multiple choice questions focusing on screening, diagnosis, treatment and follow-up of GLILD.

After April 30, 2020, data were collected and categorized for further analysis. Data were transferred and stored in an electronic database of IBM SPSS Statistics (version 23) for Windows, Armonk NY. Statistical analyses consisted of descriptive statistics and comparison of categorical data using Pearson Chi square or Fischer exact tests. A p value of < 0.05 was considered statistically significant.

## Results

### Clinicians Treating GLILD Rarely Have Access to Standardized Protocols

A total of 161 substantially completed clinician surveys were returned. Responses came from 47 different countries, most frequently Italy (n=17), followed by France, Spain, United Kingdom (each n=14) and Australia, Czech Republic, Germany, Portugal and the U.S.A. (each n=5).

The majority (n=127, 78.9%) of respondents treated adult patients and were specialized in either pulmonology (n=81, 50.3%) or immunology (n=38, 23.6%). Other specialties (n=11, 6.8%) included internal medicine and infectious diseases. The 31 responding pediatricians were specialized in immunology (n=24, 14.9%) or pulmonology (n=7, 4.3%) and two additional respondents treated both adult and pediatric immunology patients. The responses from these two subjects were analyzed in both groups for descriptive statistics but excluded from comparisons between those treating adult and pediatric patients.

Respondents treated a median of 27 (range 0 to 500) CVID disorders patients and 5 (0 to 200) GLILD patients with a large variation between respondents. Only a small proportion (n=11, 6.8%) worked at a secondary care hospital, the majority was employed at specialized settings including tertiary care hospitals (n=82, 50.9%) and/or reference centers for PID/CVID disorders (n=61, 37.9%) or ILD/sarcoidosis (n=56, 34.8%). More pediatricians worked at a PID reference center (58.1% *vs* 33.1%; p = 0.01) and/or in an academic setting (77.4% *vs* 44.9%, p=0.001) than specialists treating adults. Conversely, there were no pediatricians employed at ILD/sarcoidosis references centers, compared to 44.1% of the adult specialists (p<0.001).

Despite these specialized work environments, only 19.3% of respondents reported the availability of a dedicated GLILD protocol.

### The Diagnosis of GLILD Is Often Difficult

When asked about screening for lung disease in CVID disorders patients with no established structural lung disease (i.e. GLILD and/or airway disease), most respondents stated using pulmonary function tests at least once a year (n=110, 70.9%). Chest CT was less frequently used, with 63.2% of respondents using CT for screening in asymptomatic patients at intervals between ≥1–3 years up to every 5–10 years. Immunologists (75.4 *vs* 54.8% for pulmonologists, p=0.008) and those working at PID reference centers (83.3 *vs* 50.5%, p<0.001) reported greater use of CT screening. There were no differences between pediatricians and those caring for adults (64.3 *vs* 63.2%, p=0.548). Nearly all respondents (94.4%) admitted having at least sometimes difficulties diagnosing GLILD, with 38.3% stating that GLILD diagnosis was often difficult. These difficulties were similar between different specialties and centers.

The tests used for the evaluation of suspected GLILD are described in **Table 1**. Whilst not definitive, the majority of clinicians reported using sputum and bronchoalveolar lavage (BAL) tests, and half of them used blood investigations. The use of biopsy was much less frequent: 46 respondents (28.6%) stated that histology is required for diagnosis, but 71.4% would not routinely undertake a biopsy. Respondents were questioned on the results from biopsies from patients with suspicion of GLILD, and 46 (28.9%) out of 103 stated that alternative diagnoses had been found. Elaborating upon these alternative diagnoses, lymphoma was most frequently reported, but malignancy or lung cancer not further specified were also mentioned. Second were infections, including TB, fungal infection and one case of EBV induced lipoid pneumonia. One respondent mentioned hypersensitivity pneumonitis. Furthermore, other conditions mentioned included nonspecific interstitial pneumonia (NSIP), granulomatous diseases, sarcoidosis, lymphoproliferative disorders, post-inflammatory fibrosis and organizing pneumonia which however may be considered part of the spectrum of GLILD.

**Table 1:** Performed diagnostics in the evaluation of suspected GLILD/exclusion of other pathology.

	No. (total 161)	Percentage (%)
**Blood**	**118**	**73.3**
Aspergillus antigen blood test	80	49.7
Mycobacterium blood test	80	49.7
Beta D glucan blood test	41	25.5
Other blood tests*	33	20.5
**Sputum**	**121**	**75.2**
Bacteria	108	67.1
Mycobacteria	108	67.1
Fungal pathogens	90	55.9
Viral pathogens	43	26.7
Other sputum tests	5	3.1
**Bronchoalveolar lavage**	**129**	**80.1**
Bacteria	124	77
Mycobacteria	121	75.2
Fungal pathogens	119	73.9
Viral pathogens	87	54
Other bronchoalveolar lavage tests**	39	24.2
**Lung biopsy**	**39**	**24.2**
Bacteria	22	13.7
Mycobacteria	30	18.6
Fungal pathogens	26	16.1
Viral pathogens	17	10.6
Other biopsy tests	11	6.8

*Other blood tests include culture, autoantibody panel, beta 2 microglobulin, soluble CD25, cytology differential, Igs, procalcitonin, PCR EBV, and CMV. **Other bronchoalveolar lavage tests include next generation sequencing of pathogens, galactomannan, flow cytometry.

### Disparities in Follow-Up and Criteria for Initiation of Immunosuppressive in GLILD

Since there are no clear guidelines on how to carry out follow-up of GLILD patients, we asked whether respondents experienced difficulties in deciding follow-up. This question was filled out by 110 respondents, of which 18 (16.3%) mentioned that they did not experience difficulties at all in defining adequate follow-up for GLILD. The majority however experienced difficulties at different aspects, namely in defining the optimal time interval for follow-up (55.5%), defining the optimal monitoring method (39.1%) and how to follow-up on asymptomatic patients (43.6%) and patients that did not require current treatment (24.5%).

We asked how follow-up of asymptomatic patients not requiring therapy was carried out with regard to monitoring methods and time interval (**Table 2**). The same questions were asked for patients that did require current therapy. Questions were filled out by 102–116 respondents. As expected, the selected time intervals were on average shorter than for patients not requiring therapy. Chest X-ray was not considered to be an applicable monitoring method by 35% of the respondents. A different subset of respondents however seemed to value chest X-ray for monitoring patients requiring therapy; of the 66 respondents that used CXR in this group, almost half of them (n=32; of which 22 were adult pulmonologists) applied this modality every 3 to 4 months.

**Table 2:** Preferred monitoring time intervals for untreated and treated patients per modality.

	Asymptomatic, untreated GLILD patients		GLILD patients requiring treatment	
	1^st^ choice	2^nd^ choice	1^st^ choice	2^nd^ choice
Clinical and laboratory evaluation	3–4 monthly (n = 46, 40.4%)	6–8 monthly (n = 41, 36%)	3–4 monthly (n = 58, 50.9%)	1–2 monthly (n = 42, 36.8%)
PFT	6–8 monthly (n = 52, 44.8%)	12 monthly (n = 37, 31.9%)	3–4 monthly (n = 67, 58.3%)	6–8 monthly (n = 28, 24.3%)
CXR	12 monthly (n = 28, 26.9%)	6–8 monthly (n = 21, 19.6%)	3–4 monthly (n = 32, 31.4%)	6–8 monthly (n = 14, 13.7%)
HRCT	>12 monthly (n = 61, 53.5%)	12 monthly (n = 40, 35.1%)	6–8 monthly (n = 40, 35,4%)	12 monthly (n = 32, 28,3%)

CXR, chest X-ray; GLILD, granulomatous–lymphocytic interstitial lung disease; HRCT, high-resolution computed tomography; PFT, pulmonary function tests.

Clear-cut criteria on when to initiate immunosuppressive therapy in GLILD have not been defined and this was reflected in the dissimilar answers given to this question. A diagnosis of GLILD alone was for the majority of respondents (n=82 out of 103, 79.6%) not sufficient reason to start an immunosuppressive treatment regimen. Similarly, the presence of clinical symptoms alone (n=86, 83.5%) or deteriorating PFT (n=80, 77.6%) or HRCT findings (n=77, 74.8%) alone was usually insufficient basis for commencement of therapy. The fraction of respondents that would initiate therapy increased if there were abnormalities in two out of three of the aforementioned items but remained relatively low; (31.1% for clinical symptoms and PFT decline; up to 47.6% for HRCT and PFT deterioration). Strikingly, only 60.2% would treat “All patients with impaired lung function, clinical symptoms and worsening of CT scan”. Adult pulmonologists (75%) were most likely to initiate treatment in this patient category, followed by adult immunologists (60%) and pediatricians (12.5%).

### Therapy of GLILD꞉ Variable Use of Steroids for Remission-Induction and Maintenance Therapy

The next part of the survey included questions related to the treatment of GLILD. Respondents were questioned whether they had used glucocorticoids for remission induction and/or maintenance therapy in GLILD patients and if so, in how many patients. Of the 125 respondents that filled out this question, 82 (65.6%) had used monotherapy with glucocorticoids for remission induction. This was equally distributed between adult immunologists and pulmonologists. The majority (n=63, 77.8%) had used this regimen in 1–5 patients; ten (12.3%) and eight (9.9%) clinicians had treated 5–10 or >10 patients, respectively. Questions on dosage and tapering revealed that the commonest regimen for severe GLILD was 1mg/kg body weight (BW), as performed by 50%, but 0.5 mg/kg BW was also frequent (32/82 respondents, 39.0%). Only one respondent used a dose lower than 0.5mg/kg BW and some clinicians used more than 1mg/kg BW. The twelve responding pediatricians used significantly higher doses than clinicians treating adults only; six of them used 1mg/kg BW and the other six used >1mg/kg BW (p<0.001, Pearson Chi-square). The distribution of the tapering period of glucocorticoids was comparable between groups; most physicians (n=38, 46.3%) tapered glucocorticoids entirely or until maintenance dose within 1–3 months, but longer or more variable intervals were also reported. The experience on effectiveness of this therapy was diverse: only three respondents (3.7%) replied that nearly all patients responded; in general, respondents felt that the majority of (n=47, 58.0%) or some patients (n=27, 33.3%) responded.

The proportion of respondents that used glucocorticoids for maintenance therapy was 58 out of 124 (46.8%). Noticeably, six of them did not report using glucocorticoids for remission induction. Again, patient numbers treated by individual clinicians were small with 1–5 patients for the majority (n=47, 82.5%) of clinicians. About two-third used ≤7.5mg steroids daily and slightly under one-third used 7.6–15mg per day. Three respondents had used maintenance doses >15mg/day. The clinical response to these maintenance glucocorticoids was heterogeneous and many responded with multiple answers; complete and partial responses to maintenance glucocorticoids were noted by 20 and 62.7% of 59 respondents, respectively. A sustained response was seen by 16 (27.1%), but relapses occurred frequently as well (n=20, 33.8%).

### Therapy of GLILD꞉ Azathioprine, Rituximab and Mycophenolate Mofetil Are the Most Frequently Employed Steroid-Sparing Agents

**Figure 1:**
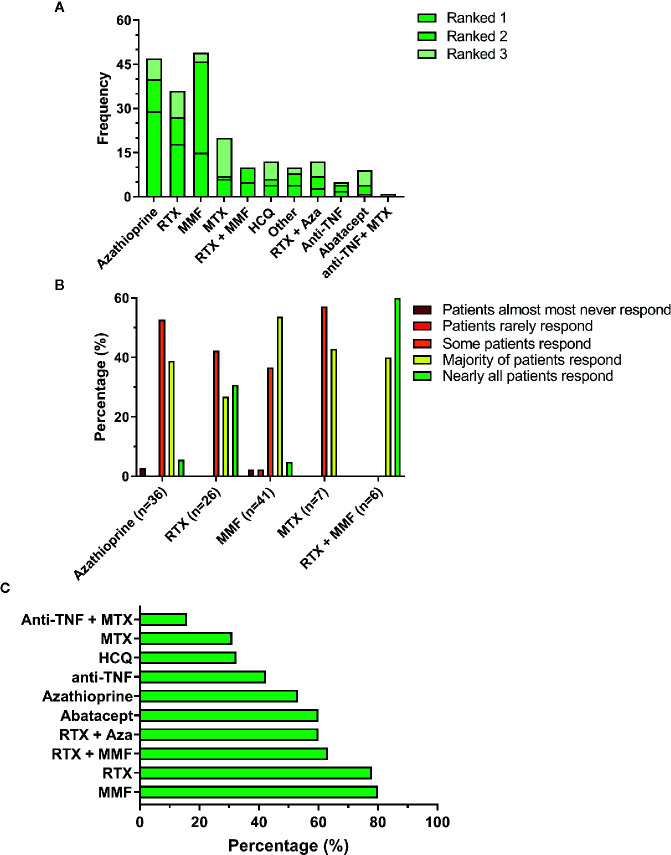
Immunosuppressive therapy in GLILD. **(A)** Top-three ranking of non-steroidal immunosuppressive drugs. **(B)** Estimated patient response rates to the 5 most highly ranked non-steroidal immunosuppressive agents according to the treating clinicians. **(C)** Percentage of respondents that would encourage the use of the non-steroidal immunosuppressive drug. Aza, azathioprine; HCQ, hydroxychloroquine; MMF, mycophenolate mofetil; MTX, methotrexate; RTX, rituximab.

Following the questions on glucocorticoid use, respondents were asked on their experience with other immunosuppressive agents for treatment of GLILD. These included both cDMARD, biologicals such as rituximab, TNF inhibitors and combinations of both. Respondents were asked to rank these drugs according to their personal practice (**Figure 1A**). **Figure 1A** shows that the three most commonly applied non-steroidal immunosuppressants were azathioprine, rituximab and mycophenolate mofetil. Noticeably, mycophenolate mofetil was frequently ranked as second choice, usually after azathioprine. Other immunosuppressants used included sirolimus, cyclosporine, and individual cases of ruxolitinib and tofacitinib.

The majority of respondents had used these drugs only in up to five and occasionally up to 10 patients. Only azathioprine (n=2), MMF (n=1), MTX (n=1) and RTX (n=1) were used in more than 10 patients. Respondents were asked to elaborate on their experience with the immunosuppressants they had ranked first and second. The cumulative responses for the top five non-steroidal agents are shown in **Figure 1B**. Noticeably, although azathioprine was the first choice for most respondents, its perceived effectiveness appeared less favorable than for other drugs; particularly the combination of rituximab with mycophenolate mofetil, but also mycophenolate mofetil alone appeared to induce a response in a larger proportion of the patients. These findings suggest that the choice of drug is not solely based on its expected clinical effectiveness but that other factors are involved; indeed, some respondents mention the costs and availability of rituximab in particular as limiting factors. These answers corresponded with the answers given to the question whether clinicians would discourage the prescription of the particular drug. Mycophenolate mofetil, rituximab and the combination of these two were less likely to be discouraged. Drugs were often discouraged for multiple reasons; usually side effects, but also other effects or ineffectiveness. Hydroxychloroquine was usually discouraged due to a lack of effect.

**Figure 2:**
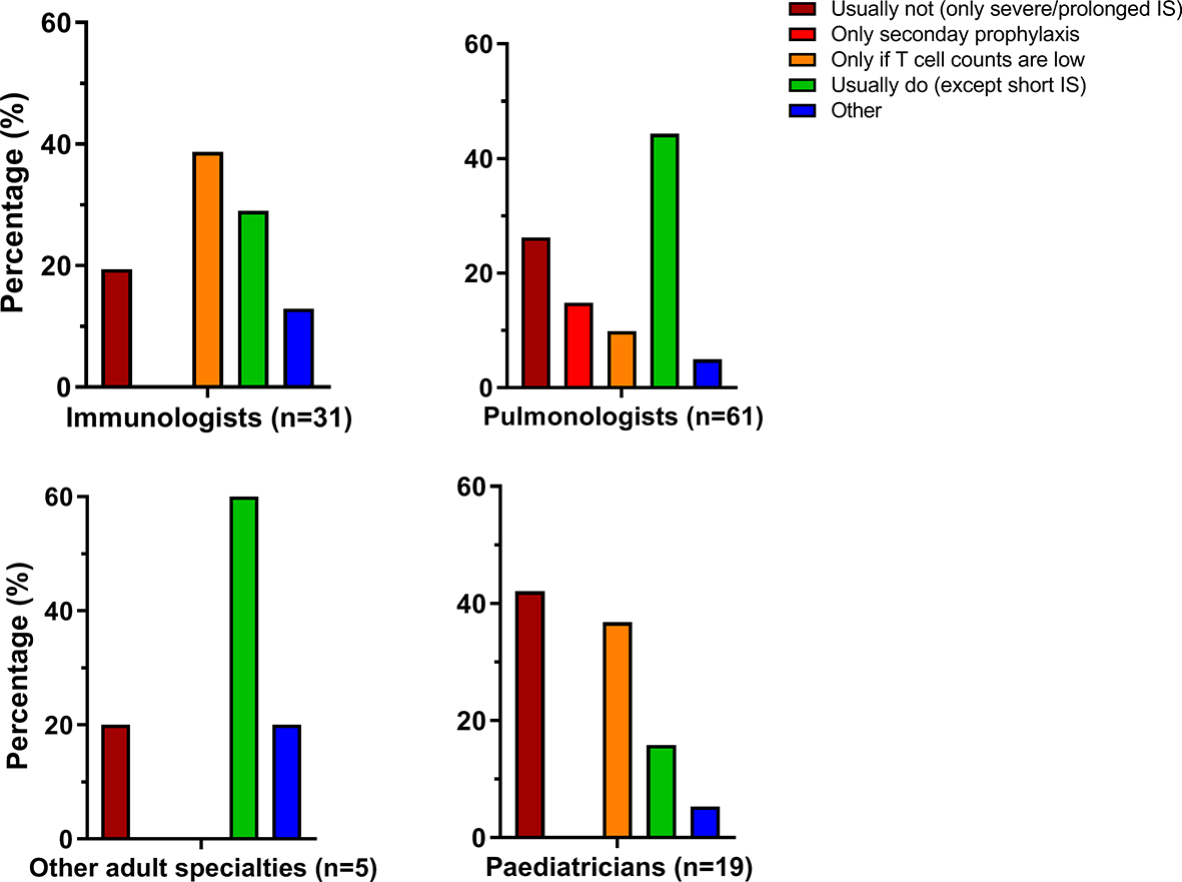
Prescription of *Pneumocytis jiroveci* pneumonia (PCP) antimicrobial prophylaxis varies within and between specialties. IS, immunosuppression.

In addition to use of immunosuppressive therapy, respondents were enquired to comment on the use of antimicrobial prophylaxis to prevent *Pneumocystis jiroveci* pneumonia (PCP). Answers were categorized into different categories as shown in **Figure 2**. The prescription of PCP prophylaxis was very heterogeneous, both within and between specialties. PCP prophylaxis appeared to be more frequently applied by adult specialists than by pediatricians, but the differences were not statistically significant. Various comments were given if the option “other” was chosen. PCP prophylaxis was often individualized and based on (combinations of) CD4^+^ T cell counts, duration of immunosuppressive therapy and combinations of immunosuppressants, particularly the combination of a DMARD with systemic glucocorticoids.

## Discussion

We present the results of an online clinician survey related to the diagnosis and management of GLILD. We received 161 responses from physicians caring for GLILD patients all over the world. The results show that there are many areas of need and uncertainty on this topic that deserve attention.

The diagnosis of GLILD is often difficult and most respondents did not have access to a GLILD protocol. CVID disorders patients were often not regularly screened for GLILD using PFT and less frequently by CT. Once GLILD was considered, the diagnosis was usually based on PFT and CT, aided by exclusion of infection *via* auxiliary blood, sputum and BAL testing. The necessity of lung biopsy remains controversial. Immunological BAL analysis was not frequently used, likely due to its uncertain value in the diagnosis of GLILD. The majority of respondents experienced difficulty defining adequate follow-up of GLILD patients. Especially, imaging monitoring would benefit from guidelines with considerable heterogeneity in the use and interval of examinations by X-ray and chest CT. Most of these findings are in line with the results of the British Lung Foundation (BLF) survey conducted among UK centers (8), which showed overall consensus regarding the original work-up of GLILD but failed to define consensus related to management strategies and the initiation of therapy in certain patient groups such as asymptomatic GLILD.

Regarding therapy, corticosteroids remain the first line of immunosuppressive induction therapy for the majority of respondents, as is common practice in literature and clinical setting (8, 15). About half of respondents of the BLF survey also use corticosteroids in low dosages to maintain remission. Of those respondents, 46% preferred non-steroidal immunosuppressive monotherapy, 13% corticosteroids alone, 21% a combination of both and 13% complete withdrawal and monitoring. The fact that in our cohort 33% uses a maintenance dose of >7.5 mg/d of prednisone may already hint towards the difficulty of choosing an alternative second line therapy.

This uncertainty is also reflected by the heterogeneous use of non-steroidal immunosuppressive agents which includes cDMARD, biologicals and combinations of both. Within this study, azathioprine, mycophenolate mofetil and rituximab were most frequently used. Indeed, for these three drugs there was 80% or greater consensus with the BLF study. However, although part of the consensus, the frequent use of azathioprine was not based on clinical evidence as a substantial fraction of the respondents did not report azathioprine as being effective in this disease. In contrast, the combination of rituximab with azathioprine first promoted by the early paper of Chase and colleagues (22) has been used successfully in several patients. The successful induction of radiological and spirometric improvement by a combination of rituximab with azathioprine or mycophenolate mofetil was confirmed in a recent extension and expansion of the original Chase study reporting retrospectively 39 GLILD patients with and without an underlying monogenetic defect (28).

In addition to a lack of evidence regarding optimal immunosuppressive therapy, the question of whether PCP prophylaxis should be employed and, if so, in which patients, remains to be answered. Antimicrobial prophylaxis was considered beneficial in a meta-analysis of a heterogeneous population of non-HIV immunocompromised patients (34). As most of these patients had both impaired humoral and cellular immune responses due to acute leukemia or organ transplantation, it remains unclear whether these findings could and should be extrapolated to all GLILD patients. The variable PCP prophylaxis strategies in our survey reflect the lack of recommendations for non-HIV immunocompromised patients. Typically, the decision is made for each case individually, including factors such as combination and duration of immunosuppressive regimen, numbers of CD4+ T lymphocytes and perhaps other elements such as age, comorbidities and physician’s preferences.

The strengths of this study include a high response rate of 161 valuable responses from 47 countries, making this the largest survey on this topic until now. Respondents represented six continents and worked in the relevant specialties of pulmonology and immunology for both pediatric and adult patients. These findings thus provide an adequate reflection of the real practice of managing GLILD in CVID disorders. Detailed responses were provided on multiple relevant subjects, including diagnosis, follow-up and therapy.

Despite our high number of responses, it still represents only a small proportion of the actual population of clinicians that take care of these patients. Hence, certain selection bias cannot be excluded. Additionally, the completeness of the answers is a limitation as it varied from ~65% to 100%. Particularly the section on therapy of GLILD was incomplete and filled out by around two-thirds of the respondents. This can be due to the length of the survey and the fact that treatment is generally carried out in multidisciplinary teams. Additionally, respondents may not feel comfortable regarding their experience with treatment of GLILD, as patient numbers were low and respondents appeared habitually reluctant to initiate therapy and treatment. Finally, this survey shows that a major limitation of current GLILD management is the lack of evidence, for which consensus is a poor substitute. There is a clear need for basic, translational and clinical research in order to eventually establish evidence-based guidelines. Basic research into the pathogenesis of GLILD should aim to elucidate the complex interplay between immune system, local micro-environment of the lungs and microbes (35) and host-microbe interactions. These findings may allow for development of targeted therapies, or optimization of the use of available drugs for improved efficacy and reduced toxicity. Since the clinic-radio-pathological picture of GLILD is very heterogeneous, the pathogenesis is probably multifaceted as well. Therapy should be optimized on the specific subtype of GLILD, perhaps eventually guided by the cellular infiltrates on biopsy, while taking into account other relevant factors such as toxicity, availability and patient preferences. Despite the pressure to see patients virtually in the current COVID-19 pandemic, this population requires face-to-face contact including clinical and diagnostic exams.

The rarity of GLILD remains an Achilles’ heel, as further dissection of this relatively small cohort into more homogenous subgroups relies on international collaboration between GLILD clinicians. Collaborative clinical studies addressing natural disease course, prognosis and treatment outcomes ought to be performed in multicenter, standardized settings. The development of an expert platform to collect data should be encouraged, as well as biobanking of biopsy specimens. Awareness, education and the availability of facilities for low-income countries are important additional topics.

The European Respiratory Society recognizes these needs and supported the launch of a Clinical Research Collaboration on GLILD, the e-GLILDnet (https://www.ersnet.org/research/e-glildnet—a-european-granulomatous-lymphocytic-interstitial-lung-disease-network; twitter: @glildnet). The e-GLILDnet aims to bring together clinicians, researchers and patients representatives from across Europe to improve the lives of those living with GLILD.

In conclusion, our survey data demonstrate an urgent need for clinical studies to provide more evidence for an international consensus regarding diagnosis and management of GLILD. The e-GLILDnet will support and facilitate this aim by supporting international collaboration, particularly on studies addressing optimal procedures for definite diagnosis and a better understanding of the pathogenesis of GLILD in order to provide individualized treatment options.

## Data Availability Statement

The raw data supporting the conclusions of this article will be made available by the authors upon request, without undue reservation.

## Ethics Statement

Ethical review and approval was not required for the study on human participants in accordance with the local legislation and institutional requirements. Written informed consent for participation was not required for this study in accordance with the national legislation and the institutional requirements.

## Author Contributions

All authors were responsible for drafting the survey. AR finalized the survey and gathered the raw data. TA and AV performed the data analyses. AV drafted the figures. AV, TA, KW, and JH drafted the paper outline. AV, TA, and AR wrote the paper, supervised by KW and JH. All authors contributed to the article and approved the submitted version.

## Funding

The e-GLILDnet Clinical research Collaboration was funded by a Clinical Research Collaboration grant of the ERS.

## Conflict of Interest

The authors declare that the research was conducted in the absence of any commercial or financial relationships that could be construed as a potential conflict of interest.
